# Flow Cytometric Cell Sorting and *In Vitro* Pre-Osteoinduction Are Not Requirements for *In Vivo* Bone Formation by Human Adipose-Derived Stromal Cells

**DOI:** 10.1371/journal.pone.0056002

**Published:** 2013-02-11

**Authors:** Yunsong Liu, Yan Zhao, Xiao Zhang, Tong Chen, Xianghui Zhao, Gui-e Ma, Yongsheng Zhou

**Affiliations:** 1 Department of Prosthodontics, School and Hospital of Stomatology, Peking University, Beijing, China; 2 Second Clinic Branch, School and Hospital of Stomatology, Peking University, Beijing, China; 3 Body Sculpture Center, Plastic Surgery Hospital, Chinese Academy of Medical Sciences, Beijing, China; University of Southern California, United States of America

## Abstract

Human adipose-derived stromal cells (hASCs) are a promising cell source for bone tissue engineering. However, before the clinical application of hASCs for the treatment of bone defects, key questions require answers, including whether pre-osteoinduction (OI) and flow cytometric cell purification are indispensible steps for *in vivo* bone formation by hASCs. In this study, hASCs were purified by flow cytometric cell sorting (FCCS). The osteogenic capabilities of hASCs and purified hASCs with or without pre-osteoinduction were examined through *in vitro* and *in vivo* experiments. We found that pre-OI enhanced the *in vitro* osteogenic capacity of hASCs. However, 8 weeks after *in vivo* implantation, there were no significant differences between hASCs and hASCs that had undergone OI (hASCs+OI) or between purified hASCs and purified hASCs+OI (*P*>0.05). Interestingly, we also found that purified hASCs had an osteogenic potential similar to that of unpurified hASCs *in vitro* and *in vivo*. These results suggest that FCCS and *in vitro* pre-OI are not requirements for *in vivo* bone formation by hASCs.

## Introduction

Many countries are faced with the problem of an aging population. An older population results in a significant increase in the number of people living with bone deformities as a consequence of bone fracture, tumor, infection and osteoporosis [1], [2], and this also has wide-ranging socio-economic impacts.

Recently, bone tissue engineering based on human adipose-derived stromal cells (hASCs) has been considered as a promising alternative to traditional treatment options for bone deformities [3]–[8]. hASCs, as a readily available, abundant supply of mesenchymal stem cells (MSCs) with minimum donor site morbidity, have been demonstrated by many studies to have osteogenic ability *in vitro* and *in vivo* [3], [4], and offer exciting opportunities to improve the quality of life of aging people suffering from bone diseases.

However, though the clinical use of hASCs for bone tissue engineering has been reported in a few studies and case reports, large human clinical trials are presently lacking [1] and many problems need to be solved before their widespread application.

Harvested populations of hASCs are a heterogeneous mixture of different cell types [9]. To study the properties of hASCs, it seems reasonable to first purify the cells. However, there is little evidence that hASCs purified by flow cytometry have higher osteogenic potential. Another problem is the necessity of *in vitro* pre-osteoinduction (OI) before *in vivo* implantation. Some researchers take it for granted that pre-OI is an indispensable step in the acquisition of osteogenic capability by hASCs [8]–[14]. However, few studies to date have provided solid evidence that *in vitro* pre-OI increases the osteogenic capability of hASCs *in vivo*. Moreover, the process of *in vitro* pre-OI prolongs the time spent by hASCs in culture and increases the risks of contamination and changes in the cells' biological behavior.

In this study, we purified hASCs by flow cytometric cell sorting (FCCS), compared the osteogenic potential of hASCs and purified hASCs, and determined the necessity of pre-OI through systematic experiments conducted *in vitro* and *in vivo*.

## Results

### hASCs express specific MSC surface markers

**Figure 1:**
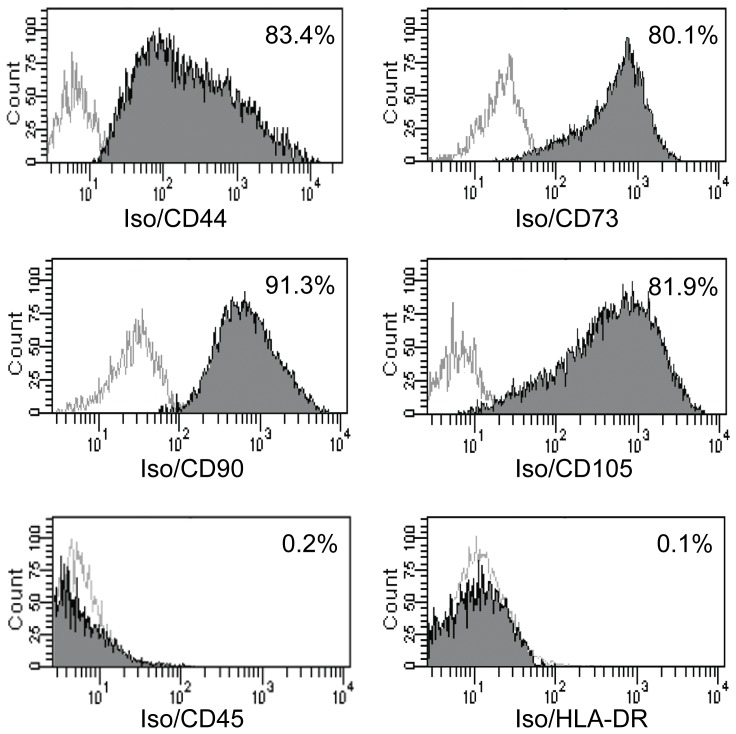
Passaged human adipose-derived stromal cells (hASCs) express mesenchymal stem cell (MSC)-specific surface markers. hASCs of the third passage (P3) expressed the MSC-specific surface markers CD44, CD73, CD90 and CD105, but did not express CD45 or HLA-DR, which are hematopoietic cell-specific markers. Isotype controls (Iso) were used in all flow cytometry experiments.

hASCs were isolated from the adipose tissues of two patients. The multi-lineage potential of the hASCs was verified (data not shown) by the methods described in previous studies [3], [4], [6]. hASCs of the third passage (P3) expressed the MSC-specific surface markers CD44, CD73, CD90 and CD105, but did not express CD45 or HLA-DR (Fig. 1), which are specific markers of hematopoietic cells.

### Purification of hASCs by flow cytometry

**Figure 2:**
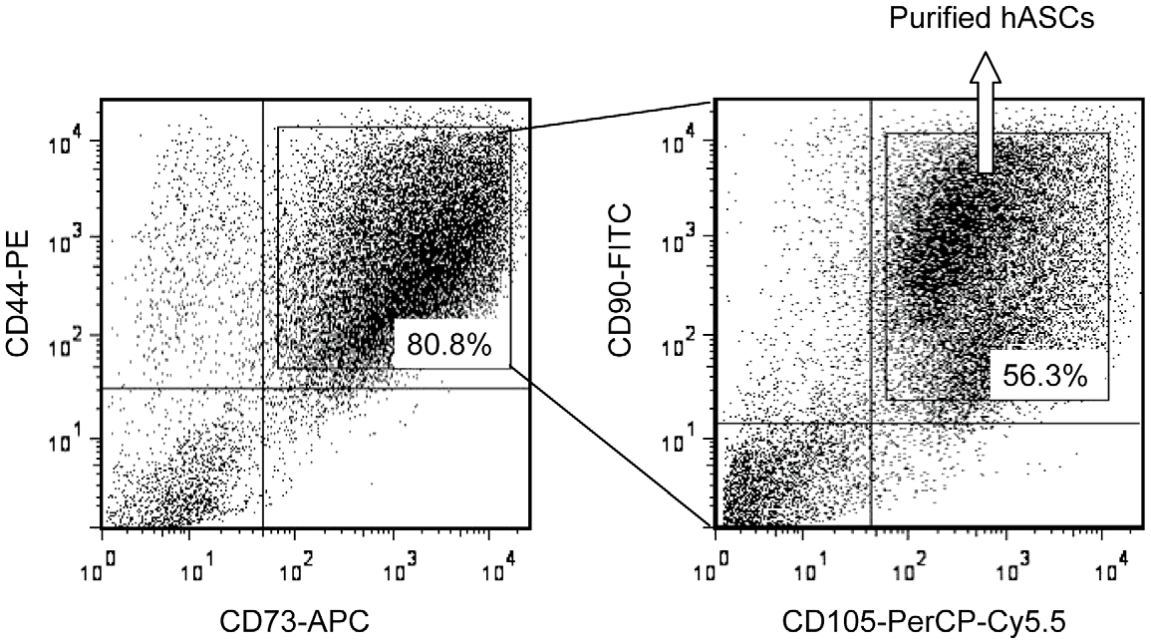
Purification of human adipose-derived stromal cells (hASCs) by flow cytometric cell sorting. Purified hASCs positive for all four of the mesenchymal stem cell-specific markers CD44, CD73, CD90 and CD105 were isolated from hASCs (third passage) by flow cytometry.

Purified hASCs positive for all four of the surface markers CD44, CD73, CD90 and CD105 were isolated from P3 hASCs by FCCS (Fig. 2). The purified hASCs were then passaged twice before the following experiments were performed. Unpurified P3 hASCs were also passaged and cultured using the same methods as for the purified hASCs.

### Comparison of proliferative capacity of hASCs and purified hASCs

**Figure 3:**
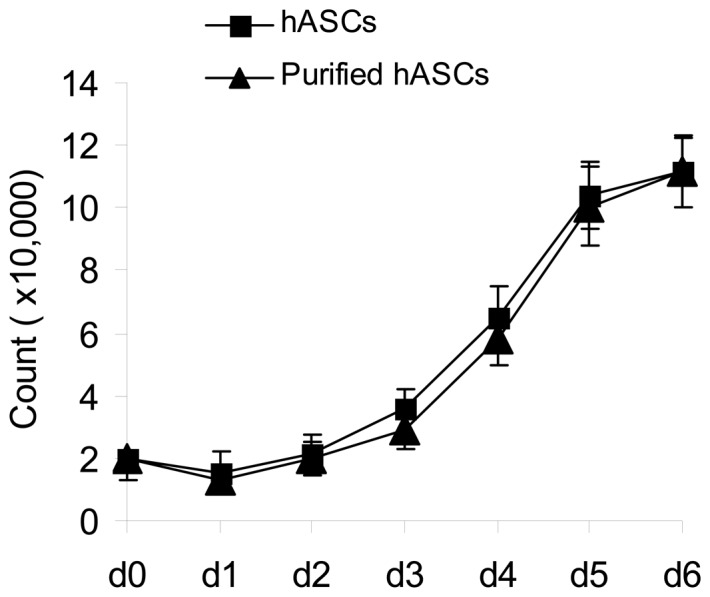
Growth curves for human adipose-derived stromal cells (hASCs) and purified hASCs. There were no significant differences in the proliferative capacities of the two cell types during day 1 (d1) to day 6 (d6) (*P*>0.05).

To compare the proliferative capacities of hASCs and purified hASCs, the two types of cell were counted daily from day 1 to day 6 (Fig. 3). These counts demonstrated no significant differences in their proliferative capacity at any time during cell proliferation (*P*>0.05).

### Comparison of osteogenesis-associated gene expression and protein secretion by hASCs and purified hASCs

**Figure 4:**
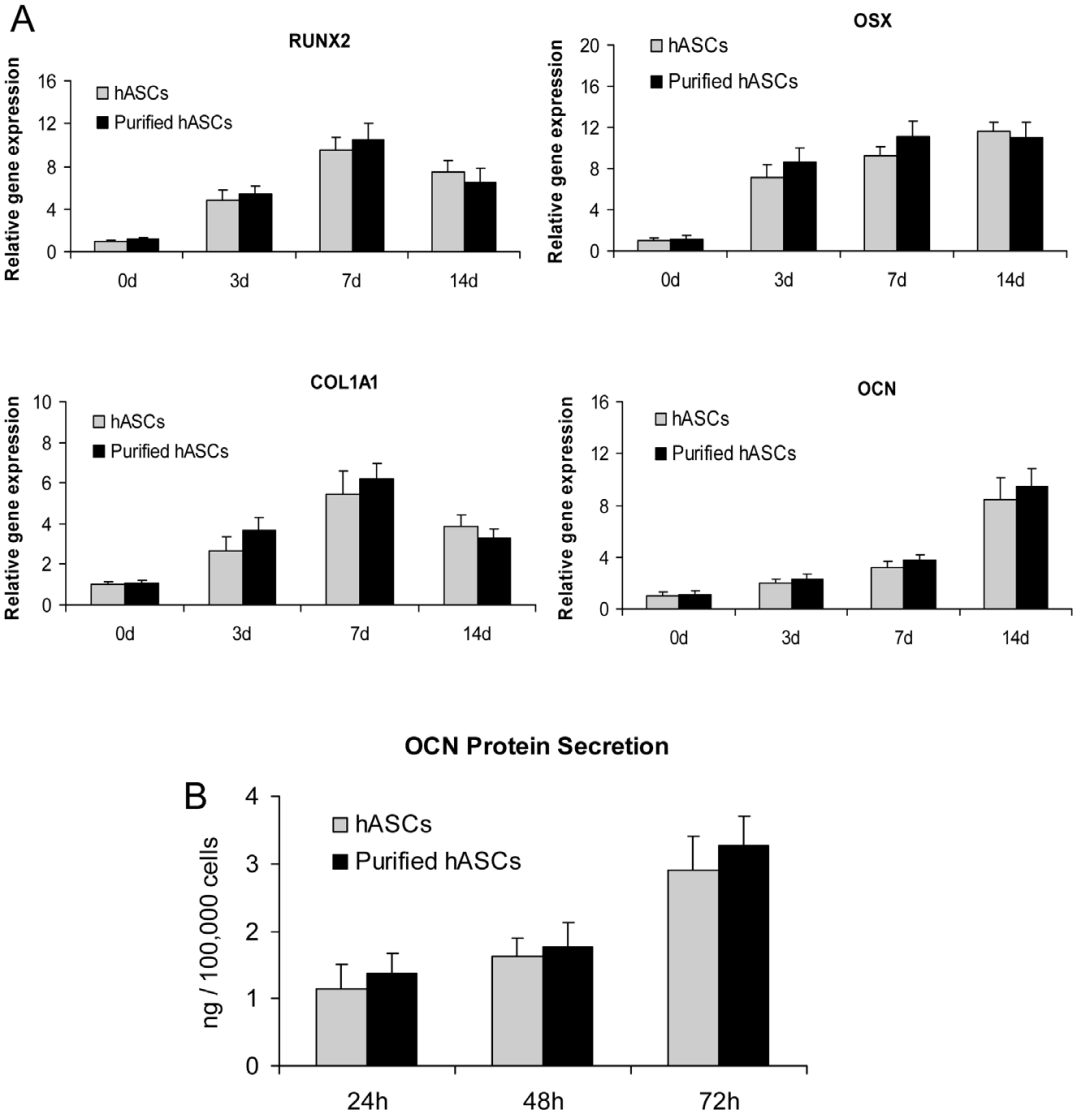
Osteogenesis-associated gene expression and protein secretion by human adipose-derived stromal cells (hASCs) and purified hASCs. A) There were no significant differences in the mRNA levels of Runt related transcription factor 2 (RUNX2), Osterix (OSX), Type I Collagen (COL1A1) or Osteocalcin (OCN) between hASCs and purified hASCs at 3, 7 or 14 days after osteogenic induction (*P*>0.05). B) There was no significant difference in OCN secretion between hASCs and purified hASCs at 24, 48 or 72 hours after osteogenic induction (*P*>0.05).

We examined the two cell types' expression of osteogenic genes such as Runt related transcription factor 2 (RUNX2), Osterix (OSX), Type I Collagen (COL1A1) and Osteocalcin (OCN) by real-time quantitative reverse transcription (qRT)-PCR. We found no significant differences (*P*>0.05) in the mRNA levels of these genes between hASCs and purified hASCs at 0, 3, 7 and 14 days after OI (Fig. 4A). The osteogenesis-associated secretion of OCN protein was also determined by radioimmunoassay. We found no significant difference (*P*>0.05) in OCN secretion between hASCs and purified hASCs at 24, 48 and 72 hours after OI (Fig. 4B).

### Comparison of osteogenic potential of hASCs and purified hASCs *in vitro*

**Figure 5:**
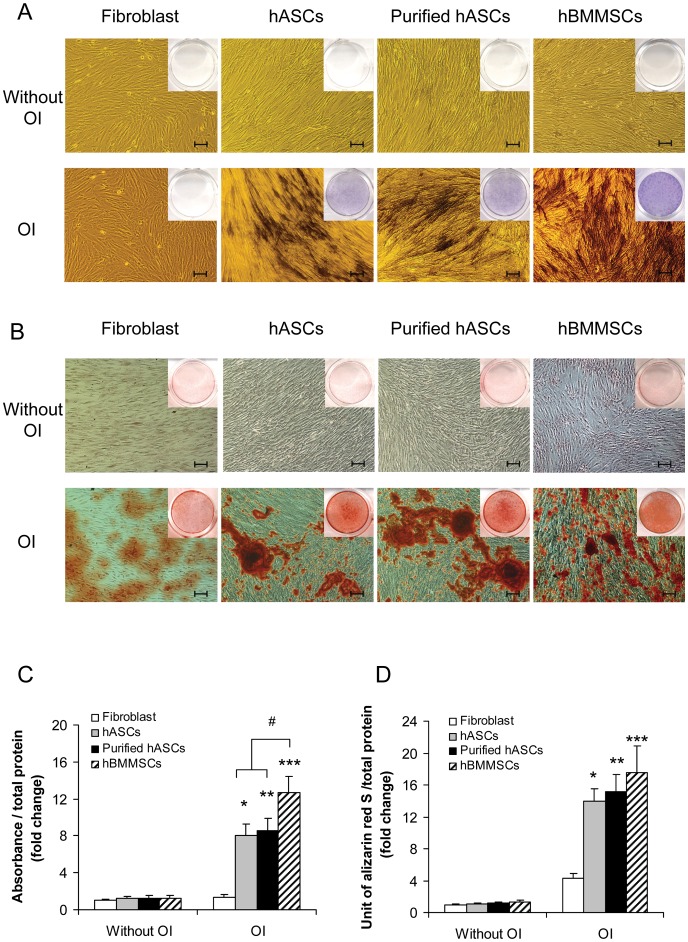
Osteogenic potential of human adipose-derived stromal cells (hASCs) and purified hASCs *in vitro*. A) hASCs and purified hASCs stained positive for alkaline phosphatase (ALP) after 7 days of osteoinduction (OI). The scale bar represents 100 µm. B) hASCs and purified hASCs were positive for Alizarin Red staining after 14 days of OI. The scale bar represents 100 µm. C) The ALP activities of hASCs, purified hASCs and hBMMSCs were significantly elevated after 7 days of OI; however, the ALP activities of both hASCs and purified hASCs were significantly lower than that of hBMMSCs (*P*<0.05). There were no significant differences between hASCs and purified hASCs or between hASCs+OI and purified hASCs+OI. D) The mineralization activities of hASCs, purified hASCs and hBMMSCs were significantly increased after 14 days of OI. There were no significant differences between hASCs and purified hASCs or between hASCs+OI and purified hASCs+OI. ^*^ *P*<0.05 compared with hASCs without OI; ^**^ *P*<0.05 compared with purified hASCs without OI; ^***^ *P*<0.05 compared with hBMMSCs without OI; ^#^ *P*<0.05 compared with hBMMSCs after 7 days of OI.

To compare the osteogenic potential of hASCs and purified hASCs *in vitro*, the cells were induced in osteogenic medium (OM) for 7 days or 14 days. Alkaline phosphatase (ALP) staining, Alizarin Red staining, ALP activity assay and quantitative Alizarin Red assays were then performed to assess the osteogenic potential of the cells. Positive results for ALP staining (Fig. 5A) and Alizarin Red staining (Fig. 5B) confirmed the osteogenic potential of both hASCs and purified hASCs. Human fibroblasts were used as negative controls and human bone marrow MSCs (hBMMSCs) as positive controls. ALP activity assay (Fig. 5C) showed the ALP activities of both hASCs and purified hASCs to be significantly elevated after 7 days of OI (*P*<0.05). However, there were no significant differences (*P*>0.05) between hASCs and purified hASCs or between hASCs that underwent OI (hASCs+OI) and purified hASCs that underwent OI (purified hASCs+OI). Quantitative Alizarin Red assay (Fig. 5D) showed the mineralization activity of both hASCs and purified hASCs to be significantly increased after 14 days of induction in OM (*P*<0.05), but there were no significant differences between hASCs and purified hASCs or between hASCs+OI and purified hASCs+OI (*P*>0.05). In addition, we found that after 7 days of OI, the ALP activities of both hASCs and purified hASCs were significant lower than that of hBMMSCs (*P*<0.05). After 14 days of OI, the mineralization activity of both hASCs and purified hASCs were slightly lower than that of hBMMSCs; however, the difference was not significant (*P*>0.05).

### Comparison of *in vivo* bone formation capability of hASCs, purified hASCs, hASCs+OI and purified hASCs+OI

**Figure 6:**
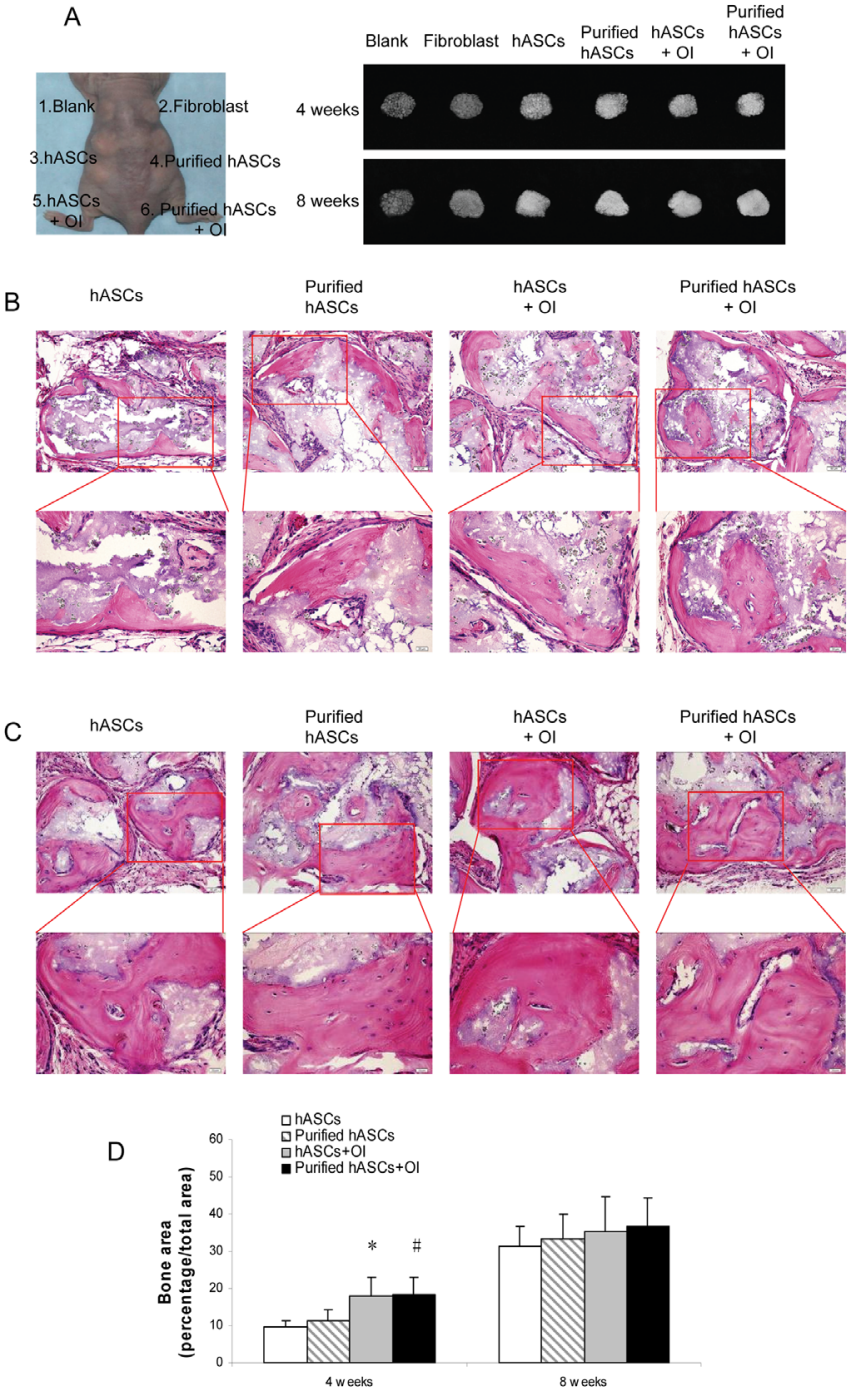
*In vivo* bone formation by human adipose-derived stromal cells (hASCs). A) Gross observation and soft X-ray examination showed that hASCs, purified hASCs, hASCs that had undergone osteoinduction (OI) (hASCs+OI) and purified hASCs+OI formed bone-like tissues of relatively higher density than blank controls and fibroblast controls. B) Hematoxylin and eosin staining showed bone-like tissues with the typical structure of osteocyte lacunae in all four groups 4 weeks after implantation. The scale bar represents 50 µm. C) Eight weeks after implantation, the area of bone formation was larger in all four groups. The scale bar represents 50 µm. D) Quantitative measurements of bone-like tissues demonstrated that, 4 weeks after implantation, the area of bone formation was significantly increased in hASCs+OI and purified hASCs+OI compared with hASCs and purified hASCs without OI. However, 8 weeks after implantation there were no significant differences among the four groups. ^*^ *P*<0.05 compared with hASCs without OI; ^#^ *P*<0.05 compared with purified hASCs without OI.

To compare *in vivo* bone formation capabilities and to determine the function of *in vitro* pre-OI, hASCs, purified hASCs, hASCs+OI and purified hASCs+OI were transplanted subcutaneously into nude mice along with β-tricalcium phosphate (β-TCP). Blank controls and fibroblast controls were used in this experiment. Gross observation and soft X-ray examination showed that hASCs, purified hASCs, hASCs+OI and purified hASCs+OI could all form bone-like tissues with a relatively higher density than blank controls and fibroblast controls (Fig. 6A).

Following hematoxylin and eosin staining (data for blank controls and fibroblast controls are not shown), eosinophilic bone-like tissues with the typical structure of osteocyte lacunae were observed in hASCs, purified hASCs, hASCs+OI and purified hASCs+OI 4 weeks after implantation (Fig. 6B). Quantitative measurements demonstrated that the area of bone formation was significantly increased in hASCs+OI and purified hASCs+OI compared with hASCs and purified hASCs without pre-OI (*P*<0.05); there were no significant differences between hASCs and purified hASCs or between hASCs+OI and purified hASCs+OI (Fig. 6D, *P*>0.05). Eight weeks after implantation, the areas of bone formation were markedly larger in hASCs, purified hASCs, hASCs+OI and purified hASCs+OI (Fig. 6C); interestingly, there were no significant differences among the four groups (Fig. 6D, *P*>0.05).

## Discussion

Like bone marrow MSCs, ASCs have extensive osteogenic capacity both *in vitro* and *in vivo* in several species, greatly enhancing the healing of bone defects [6]–[8], [10]–[12]. The use of scaffolds in combination with hASCs provides a valuable tool for bone regeneration [13]–[16], especially in patients with complex anatomic defects. However, before the extensive clinical application of hASCs, a series of standard protocols should be established and many questions should be definitively answered, including whether flow cytometric cell purification and *in vitro* pre-OI are requirements for *in vivo* bone formation by hASCs.

As reported in other studies, hASCs are not a pure cell population. Instead, they comprise a mixture of different cell types including MSCs, adipose progenitor cells, endothelial progenitor cells and fibroblasts [1], [9], [17]. Previous studies have made efforts to purify hASCs by flow cytometry [18], [19]; CD44, CD73, CD90 and CD105 are well accepted markers identifying MSCs [19]–[23] and can be used to purify hASCs. However, for the purpose of clinical use of hASCs, FCCS is a complicated and expensive technique that is difficult to apply extensively. Moreover, there are no solid data from previous studies supporting the contention that purified hASCs have better osteogenic capability *in vivo*. In the present study, purified hASCs positive for all four of the surface markers CD44, CD73, CD90 and CD105 were isolated by flow cytometry.

In subsequent *in vitro* experiments, we found no notable differences in osteogenic gene expression or protein secretion between hASCs and purified hASCs. Consistently, ALP activity and quantitative Alizarin Red assays showed no significant differences between the two cell types. In *in vivo* experiments, both hASCs and purified hASCs formed ectopic bone structures under the skin of nude mice and quantitative measurements showed no significant differences between hASCs and purified hASCs with or without *in vitro* pre-OI at 4 weeks or 8 weeks after transplantation.

These results suggest that unpurified hASCs have similar osteogenic potential to hASCs purified by FCCS. This may be because MSCs in unpurified hASCs can out-compete other cell populations over time under *in vitro* culture conditions [17]. Considering the cost and complicated procedure required for FCCS, the finding that flow cytometric cell purification is not a requirement for *in vivo* bone formation by hASCs will facilitate the clinical application of hASCs in the future.

Pre-OI has been regarded as an indispensable step for *in vivo* bone formation by hASCs [24]–[32]. The *in vitro* experiments in this study demonstrated that OI could indeed increase the osteogenic capacity of hASCs. However, our *in vivo* experiments showed that non-induced hASCs could also form bone structures when transplanted subcutaneously into nude mice. Interestingly, though the area of bone formation was larger for hASCs+OI than for hASCs 4 weeks after transplantation, there was no significant difference between these groups after 8 weeks. Similar results were obtained for purified hASCs and purified hASCs+OI. These findings suggest that pre-OI is not a requirement for *in vivo* bone formation by hASCs.

It has been reported that osteogenic signaling molecules can accumulate in the region of bone defects by paracrine pathways [33]. This may be one reason why hASCs can transform into osteogenic cells *in vivo* without pre-OI. In addition, like hBMMSCs, hASCs can secrete extracellular matrix, which supports a microenvironment that is suitable for the growth of new bone [34], [35]. It is well known that dexamethasone in classical OM decreases the proliferative activity or differentiation efficiency of MSCs [36], [37]; meanwhile, the process of *in vitro* pre-OI is time consuming and can cause problems including contamination and changes in cell behavior. Niemeyer et al. found that undifferentiated MSCs are candidates for non-autologous cell transplantation, whereas osteogenically induced MSCs seem to be eliminated by the host's immune system when ASCs or BMMSCs are used in bone tissue engineering [38]. Therefore, omission of pre-OI should markedly improve the safety, efficiency and universality of the clinical application of hASCs.

β-TCP was used as a scaffold material in our *in vivo* experiments because of its suitable mechanical properties and good biological compatibility. Moreover, the ratio of calcium to phosphate in β-TCP is similar to that in natural bone tissues, and it is widely used as a scaffold in bone tissue engineering [39], [40]. Human fibroblasts were used as negative controls in our *in vitro* and *in vivo* experiments. However, in the Alizarin Red assay, we found weakly positive staining of the fibroblasts after OI. Similar results were reported by Locke et al. [17]. The cause of this false positive result may be the lack of specificity of this staining method. hBMMSCs were utilized as positive controls in our *in vitro* study. hBMMSCs are considered to be an effective source of MSCs and have been widely used in bone regenerative medicine for many years [24], [30], [33], [38], [41], [42]. In our experiments, we found that the ALP activities of both hASCs and purified hASCs were significant lower than that of hBMMSCs after 7 days of OI; however, there were no significant differences in the mineralization capacities of hASCs and hBMMSCs after 14 days of OI. Regarding *in vivo* osteogenic capability, Cowan et al. [33] demonstrated that ASCs displayed more bone formation than BMMSCs at an early stage of *in vivo* bone regeneration. However, Sakaguchi et al. [43] made opposite findings. Further investigations are needed in the future to provide a definitive answer to this question.

In summary, through systematic *in vitro* and *in vivo* experiments, we can draw the conclusion that neither FCCS nor *in vitro* pre-OI is an indispensable step for *in vivo* bone formation by hASCs. These findings will accelerate the application of hASCs from bench to bed.

## Materials and Methods

All materials were purchased from Sigma-Aldrich (St. Louis, MO, USA) unless otherwise stated.

### Ethics statement and hASCs harvest

hASCs were isolated according to previously published methods [44], [45]. Briefly, human adipose tissues were obtained with written informed consent from two healthy patients who were undergoing liposuction for esthetic reasons in the plastic surgery hospital affiliated to the Chinese Medical Academy. This study was approved by the Ethics Committee of the Peking University Health Science Center, Beijing, China (IRB00001052-06032) and all animal experiments were performed in accordance with the approved animal protocol of the Peking University Health Science Center. All surgery was performed under sodium pentobarbital anesthesia and all efforts were made to minimize suffering.

The liposuction tissue was washed at least three times with equal volumes of phosphate-buffered saline (PBS) and then digested with 0.075% type I collagenase for 60 min at 37°C with intermittent shaking. The floating adipocytes were separated from the stromal cells by centrifugal force (300×*g*) for 10 min and a cell pellet was obtained. The pellet was resuspended in 160 mM NH_4_Cl and incubated at room temperature for 10 min to lyse contaminating red blood cells. The stromal cells were then collected by centrifugation, filtered through a 100 µm nylon mesh to remove cellular debris and cultured in maintenance medium (Dulbecco's Modified Eagle Medium containing 10% fetal bovine serum FBS, 100 U/ml penicillin G and 100 µg/ml streptomycin) at 37°C in an incubator with an atmosphere comprising 95% air, 5% CO_2_ and 100% relative humidity. P3 cells were used for FCCS. All experiments *in vitro* were repeated three times using hASCs from two patients.

A human fibroblast cell line (ATCC, Manassas, VA, USA) was used as a control and cultured in the same maintenance medium as the hASCs.

### Flow cytometric analysis and cell sorting

Single hASCs were harvested using trypsin and, following neutralization in 10% serum, resuspended in fluorescence activated cell sorting (FACS) buffer (PBS + 0.5% bovine serum albumin) at a concentration of 10^6^ cells/mL. For each antibody used, 10^5^ cells were stained. Antibodies to CD45-APC, HLA-DR-FITC, CD44-PE, CD73-APC, CD90-FITC and CD105-PerCP-Cy5.5 and isotype control were obtained from BD Biosciences (San Diego, CA, USA). Before staining, the cells were treated with blocking buffer (BD Biosciences) for 10 min. Staining with the appropriate dilution of the antibody was performed for 30 min on ice in blocking buffer. After two washes in FACS buffer, the cells were resuspended in FACS buffer. Analysis and cell sorting were conducted using a FACSAriaII Flow Cytometer with CellQuest software (BD Biosciences).

To obtain purified hASCs, cells were simultaneously stained with CD44-PE, CD73-APC, CD90-FITC and CD105-PerCP-Cy5.5. Strict sorting gates were set based on the isotype control, and the population that was positive for all four of the MSC-specific surface markers (purified hASCs) was sorted into ice-cold 5 mL FACS tubes containing 10% FBS in PBS.

### Growth curve

hASCs and purified hASCs were seeded in 24-well plates at a density of 1×10^4^/cm^2^ and four wells of cells were harvested daily from day 1 to day 6. The cells were counted using Vi-cell™ (Beckman Coulter, Germany) and growth curves were obtained according to cell number (mean±standard deviation).

### Cell differentiation assay

hASCs, purified hASCs and fibroblasts were grown in osteogenesis inducing medium [3] containing 100 µM ascorbic acid, 2 mM β-glycerophosphate and 10 nM dexamethasone. For ALP staining, after 7 days of induction, cells were fixed with 70% ethanol and incubated with a solution of 0.25% naphthol AS-BI phosphate and 0.75% Fast Blue BB dissolved in 0.1 M Tris buffer (pH 9.3). ALP activity assays were performed using an ALP kit according to the manufacturer's protocol and normalized to total protein concentrations. To assess mineralization, cells were induced for 14 days, fixed with 70% ethanol and stained with 2% Alizarin Red. To quantitatively determine calcium mineral density, Alizarin Red was destained with 10% cetylpyridinium chloride in 10 mM sodium phosphate for 30 min at room temperature. The concentration was determined by measurement of absorbance at 562 nm on a multiplate reader using a standard calcium curve prepared with the same solution. The final calcium levels in each group were normalized to the total protein concentrations obtained from duplicate plates.

### Real-time qRT-PCR

Total RNA was extracted according to the manufacturer's protocol (Invitrogen, Carlsbad, CA, USA). Two microgram aliquots of RNA were synthesized using random hexamers and reverse transcriptase according to the manufacturer's protocol (Invitrogen). Real-time quantitative PCR reactions were performed using a QuantiTect SYBR Green PCR kit (Qiagen, Venlo, Netherlands) and the Icycler iQ Multi-color Real-time PCR Detection System. The primers were synthesized by Invitrogen and are listed in Table 1. To control for variability in amplification owing to differences in starting mRNA concentration, glyceraldehyde 3-phosphate dehydrogenase was used as an internal standard.

**Table 1:** Sequences of primers used for real-time PCR.

Gene	Forward primer	Reverse primer
*RUNX2*	TCTTAGAACAAATTCTGCCCTTT	TGCTTTGGTCTTGAAATCACA
*OSX*	CCTCCTCAGCTCACCTTCTC	GTTGGGAGCCCAAATAGAAA
*COL1A1*	TTGCTCCCCAGCTGTCTTAT	TCCCCATCATCTCCATTCTT
*OCN*	CACTCCTCGCCCTATTGGC	CCCTCCTGCTTGGACACAAAG
*GAPDH*	ATGGGGAAGGTGAAGGTCG	GGGGTCATTGATGGCAACAATA

### Detection of osteocalcin secretion

For the quantitative determination of OCN protein secretion by hASCs and purified hASCs, an osteocalcin ^125^I radioimmunoassay kit (Chinese Institute of Atomic Energy, Beijing, China) was used. This assay is based on a competitive reaction among ^125^I human OCN, sample (culture medium) OCN, and rabbit antihuman OCN antibodies (polyclonal). After incubation at 4°C for 20 h, separation solution (a complex of donkey antirabbit antibodies, rabbit serum, and polyethylene glycol) was added to each reaction tube. After incubation for 15 min at room temperature and centrifugation for 15 min at 4°C, the counts per minute (cpm) values of the deposits were determined by counting machine. The OCN contents was then calculated according to a standard curve.

### Subcutaneous transplantation in nude mice

Transplantation in nude mice was performed as described previously [45]. Briefly, 2×10^6^ cells were mixed with 40 mg of β-TCP carrier (Bicon, Boston, MA, USA) and then transplanted subcutaneously into the dorsa of 8-week-old nude mice. Six transplantation sites were prepared in each mouse and transplanted with six groups of cells: (1) β-TCP only (blank control); (2) β-TCP + human fibroblasts (fibroblast control); (3) β-TCP + hASCs (hASCs); (4) β-TCP + purified hASCs (purified hASCs); (5) β-TCP + hASCs with OI (hASCs+OI); and (6) β-TCP + purified hASCs with OI (purified hASCs +OI). Sixteen nude mice were used in this experiment; samples were collected from eight mice at 4 weeks after transplantation and from the remaining eight after 8 weeks. Sample preparation and histomorphometric analysis were performed as described previously [46]. For quantification of bone-like tissue, 10 images of each sample were taken randomly (Olympus, Tokyo, Japan) and SPOT 4.0 software (Diagnostic Instruments, Sterling Heights, MI, USA) was used to measure the area of new bone formation versus total area.

### Statistical analysis

Data are expressed as the mean±standard deviation and analyzed using SPSS software. Student' *t*-test or one-way analysis of variance followed by Fisher' least significant difference test was performed. For all tests, statistical significance was accepted at *P*-values lower than 0.05.

## References

[1] Levi B, Longaker MT (2011) Concise review: adipose-derived stromal cells for skeletal regenerative medicine. Stem Cells 29 (4) 576–582. 21305671 10.1002/stem.612 PMC3323288

[2] Li X, Yao J, Wu L, Jing W, Tang W, et al (2010) Osteogenic induction of adipose-derived stromal cells: not a requirement for bone formation in vivo. Artif Organs 34 (1) 46–54. 19821812 10.1111/j.1525-1594.2009.00795.x

[3] Liu Y, Zhou Y, Feng H, Ma GE, Ni Y (2008) Injectable tissue-engineered bone composed of human adipose-derived stromal cells and platelet-rich plasma. Biomaterials 29 (23) 3338–3345. 18485475 10.1016/j.biomaterials.2008.04.037

[4] Zhou Y, Ni Y, Liu Y, Zeng B, Xu Y, et al (2010) The role of simvastatin in the osteogenesis of injectable tissue-engineered bone based on human adipose-derived stromal cells and platelet-rich plasma. Biomaterials 31 (20) 5325–5335. 20381859 10.1016/j.biomaterials.2010.03.037

[5] Levi B, James AW, Nelson ER, Vistnes D, Wu B, et al (2010) Human adipose derived stromal cells heal critical size mouse calvarial defects. PLoS One 5 (6) e11177. 20567510 10.1371/journal.pone.0011177 PMC2887361

[6] Zuk PA, Zhu M, Mizuno H, Huang J, Futrell JW, et al (2001) Multilineage cells from human adipose tissue: implications for cell-based therapies. Tissue Eng 7 (2) 211–28. 11304456 10.1089/107632701300062859

[7] Behr B, Tang C, Germann G, Longaker MT, Quarto N (2011) Locally applied vascular endothelial growth factor A increases the osteogenic healing capacity of human adipose-derived stem cells by promoting osteogenic and endothelial differentiation. Stem Cells 29 (2) 286–296. 21732486 10.1002/stem.581 PMC3400547

[8] Hong JM, Kim BJ, Shim JH, Kang KS, Kim KJ, et al (2012) Enhancement of bone regeneration through facile surface functionalization of solid freeform fabrication-based three-dimensional scaffolds using mussel adhesive proteins. Acta Biomater 8 (7) 2578–2586. 22480947 10.1016/j.actbio.2012.03.041

[9] Amos PJ, Bailey AM, Shang H, Katz AJ, Lawrence MB, et al (2008) Functional binding of human adipose-derived stromal cells: effects of extraction method and hypoxia pretreatment. Ann Plast Surg 60 (4) 437–444. 18362576 10.1097/SAP.0b013e318095a771 PMC2829884

[10] Monaco E, Bionaz M, Hollister SJ, Wheeler MB (2011) Strategies for regeneration of the bone using porcine adult adipose-derived mesenchymal stem cells. Theriogenology 75 (8) 1381–1399. 21354606 10.1016/j.theriogenology.2010.11.020

[11] Lu Z, Roohani-Esfahani SI, Wang G, Zreiqat H (2012) Bone biomimetic microenvironment induces osteogenic differentiation of adipose tissue-derived mesenchymal stem cells. Nanomedicine 8 (4) 507–515. 21839050 10.1016/j.nano.2011.07.012

[12] Correia C, Bhumiratana S, Yan LP, Oliveira AL, Gimble JM, et al (2012) Development of silk-based scaffolds for tissue engineering of bone from human adipose-derived stem cells. Acta Biomater 8 (7) 2483–2492. 22421311 10.1016/j.actbio.2012.03.019 PMC3367114

[13] Ying X, Cheng S, Wang W, Lin Z, Chen Q, et al (2012) Effect of lactoferrin on osteogenic differentiation of human adipose stem cells. Int Orthop 36 (3) 647–653. 21713451 10.1007/s00264-011-1303-x PMC3291782

[14] Cheng NC, Chang HH, Tu YK, Young TH (2012) Efficient transfer of human adipose-derived stem cells by chitosan/gelatin blend films. J Biomed Mater Res B Appl Biomater 100 (5) 1369–1377. 22566407 10.1002/jbm.b.32706

[15] Santo VE, Duarte AR, Popa EG, Gomes ME, Mano JF, et al (2012) Enhancement of osteogenic differentiation of human adipose derived stem cells by the controlled release of platelet lysates from hybrid scaffolds produced by supercritical fluid foaming. J Control Release 162 (1) 19–27. 22698936 10.1016/j.jconrel.2012.06.001

[16] Kang JM, Han M, Park IS, Jung Y, Kim SH (2012) Adhesion and differentiation of adipose-derived stem cells on a substrate with immobilized fibroblast growth factor. Acta Biomater 8 (5) 1759–1767. 22285427 10.1016/j.actbio.2012.01.005

[17] Locke M, Feisst V, Dunbar PR (2011) Concise Review: Human Adipose-Derived Stem Cells: Separating Promise from Clinical Need. Stem Cells 29 (3) 404–411. 21425404 10.1002/stem.593

[18] Rada T, Reis RL, Gomes ME (2011) Distinct stem cells subpopulations isolated from human adipose tissue exhibit different chondrogenic and osteogenic differentiation potential. Stem Cell Rev 7 (1) 64–76. 20396979 10.1007/s12015-010-9147-0

[19] Al Battah F, De Kock J, Vanhaecke T, Rogiers V (2011) Current status of human adipose-derived stem cells: differentiation into hepatocyte-like cells. ScientificWorldJournal 11: 1568–1581. 22224071 10.1100/tsw.2011.146 PMC3201629

[20] Zhou Y, Yan Z, Zhang H, Lu W, Liu S, et al (2011) Expansion and delivery of adipose-derived mesenchymal stem cells on three microcarriers for soft tissue regeneration. Tissue Eng Part A 17 (23–24) 2981–2997. 21875329 10.1089/ten.tea.2010.0707

[21] Schaffler A, Buchler C (2007) Concise review: adipose tissue-derived stromal cells–basic and clinical implications for novel cell-based therapies. Stem Cells 25 (4) 818–827. 17420225 10.1634/stemcells.2006-0589

[22] Gimble JM, Katz AJ, Bunnell BA (2007) Adipose-derived stem cells for regenerative medicine. Circ Res 100 (9) 1249–1260. 17495232 10.1161/01.RES.0000265074.83288.09 PMC5679280

[23] McIntosh K, Zvonic S, Garrett S, Mitchell JB, Floyd ZE, et al (2006) The immunogenicity of human adipose-derived cells: temporal changes in vitro. Stem Cells 24 (5) 1246–1253. 16410391 10.1634/stemcells.2005-0235

[24] Yuan J, Cui L, Zhang WJ, Liu W, Cao Y (2007) Repair of canine mandibular bone defects with bone marrow stromal cells and porous β-tricalcium phosphate. Biomaterials 28 (6) 1005–1013. 17092556 10.1016/j.biomaterials.2006.10.015

[25] Szpalski C, Nguyen PD, Cretiu Vasiliu CE, Chesnoiu-Matei I, Ricci JL, et al (2012) Bony engineering using time-release porous scaffolds to provide sustained growth factor delivery. J Craniofac Surg 23 (3) 638–644. 22565873 10.1097/SCS.0b013e31824db8d4

[26] Li X, Liu H, Niu X, Fan Y, Feng Q, et al (2011) Osteogenic differentiation of human adipose-derived stem cells induced by osteoinductive calcium phosphate ceramics. J Biomed Mater Res B Appl Biomater 97 (1) 10–19. 21290570 10.1002/jbm.b.31773

[27] Csaki C, Matis U, Mobasheri A, Ye H, Shakibaei M (2007) Chondrogenesis, osteogenesis and adipogenesis of canine mesenchymal stem cells: a biochemical, morphological and ultrastructural study. Histochem Cell Biol 128 (6) 507–520. 17922135 10.1007/s00418-007-0337-z

[28] Niemeyer P, Kornacker M, Mehlhorn A, Seckinger A, Vohrer J, et al (2007) Comparison of immunological properties of bone marrow stromal cells and adipose tissue-derived stem cells before and after osteogenic differentiation in vitro. Tissue Eng 13 (1) 111–121. 17518585 10.1089/ten.2006.0114

[29] Hofmann S, Hagenmuller H, Koch AM, Muller R, Vunjak-Novakovic G, et al (2007) Control of in vitro tissue-engineered bone-like structures using human mesenchymal stem cells and porous silk scaffolds. Biomaterials 28 (6) 1152–1162. 17092555 10.1016/j.biomaterials.2006.10.019

[30] Endres M, Hutmacher DW, Salgado AJ, Kaps C, Ringe J, et al (2003) Osteogenic induction of human bone marrow-derived mesenchymal progenitor cells in novel synthetic polymer-hydrogel matrices. Tissue Eng 9 (4) 689–702. 13678447 10.1089/107632703768247386

[31] Kim HP, Ji YH, Rhee SC, Dhong ES, Park SH, et al (2012) Enhancement of bone regeneration using osteogenic-induced adipose-derived stem cells combined with demineralized bone matrix in a rat critically-sized calvarial defect model. Curr Stem Cell Res Ther 7 (3) 165–172. 22329583 10.2174/157488812799859847

[32] Dudas JR, Marra KG, Cooper GM, Penascino VM, Mooney MP, et al (2006) The osteogenic potential of adipose-derived stem cells for the repair of rabbit calvarial defects. Ann Plast Surg 56 (5) 543–548. 16641633 10.1097/01.sap.0000210629.17727.bd

[33] Cowan CM, Shi Y-Y, Aalami OO, Chou Y-F, Mari C, et al (2004) Adipose-derived adult stromal cells heal critical-size mouse calvarial defects. Nat Biotechnol 22 (5) 560–567. 15077117 10.1038/nbt958

[34] Miyahara Y, Nagaya N, Kataoka M, Yanagawa B, Tanaka K, et al (2006) Monolayered mesenchymal stem cells repair scarred myocardium after myocardial infarction. Nat Med 12 (4) 459–465. 16582917 10.1038/nm1391

[35] Phinney DG (2007) Biochemical heterogeneity of mesenchymal stem cell populations: clues to their therapeutic efficacy. Cell Cycle 6 (23) 2884–2889. 18000405 10.4161/cc.6.23.5095

[36] Cooper MS, Hewison M, Stewart PM (1999) Glucocorticoid activity, inactivity and the osteoblast. J Endocrinol 163 (2) 159–164. 10556763 10.1677/joe.0.1630159

[37] Zuk PA, Zhu M, Ashjian P, De Ugarte DA, Huang JI, et al (2002) Human adipose tissue is a source of multipotent stem cells. Mol Biol Cell 13 (12) 4279–4295. 12475952 10.1091/mbc.E02-02-0105 PMC138633

[38] Niemeyer P, Vohrer J, Schmal H, Kasten P, Fellenberg J, et al (2008) Survival of human mesenchymal stromal cells from bone marrow and adipose tissue after xenogenic transplantation in immunocompetent mice. Cytotherapy 10 (8) 784–795. 18951271 10.1080/14653240802419302

[39] Coelho PG, Coimbra ME, Ribeiro C, Fancio E, Higa O, et al (2009) Physico/chemical characterization and preliminary human histology assessment of a β-TCP particulate material for bone augmentation. Mater Sci Eng C Mater Biol Appl: C 29 (7) 2085–2091.

[40] Morelhao SL, Coelho PG, Honnicke MG (2010) Synchrotron X-ray imaging via ultra-small-angle scattering: principles of quantitative analysis and application in studying bone integration to synthetic grafting materials. Eur Biophys J 39 (5) 861–865. 19784835 10.1007/s00249-009-0541-y

[41] Xu J, Zheng Z, Fang D, Gao R, Liu Y, et al (2012) Mesenchymal stromal cell-based treatment of jaw osteoradionecrosis in Swine. Cell Transplant 21 (8) 1679–1686. 22469112 10.3727/096368911X637434

[42] Liu Y, Wang L, Kikuiri T, Akiyama K, Chen C, et al (2011) Mesenchymal stem cell-based tissue regeneration is governed by recipient T lymphocytes via IFN-gamma and TNF-alpha. Nat Med 17 (12) 1594–1601. 22101767 10.1038/nm.2542 PMC3233650

[43] Sakaguchi Y, Sekiya I, Yagishita K, Muneta T (2005) Comparison of human stem cells derived from various mesenchymal tissues: superiority of synovium as a cell source. Arthritis Rheum 52 (8) 2521–2529. 16052568 10.1002/art.21212

[44] Zhou YS, Liu YS, Tan JG (2006) Is 1, 25-dihydroxyvitamin D3 an ideal substitute for dexamethasone for inducing osteogenic differentiation of human adipose tissue-derived stromal cells in vitro? Chin Med J (Engl) 119 (15) 1278–1286. 16919187

[45] Ge W, Shi L, Zhou Y, Liu Y, Ma GE, et al (2011) Inhibition of osteogenic differentiation of human adipose-derived stromal cells by retinoblastoma binding protein 2 repression of RUNX2-activated transcription. Stem Cells 29 (7) 1112–1125. 21604327 10.1002/stem.663

[46] Fan Z, Yamaza T, Lee JS, Yu J, Wang S, et al (2009) BCOR regulates mesenchymal stem cell function by epigenetic mechanisms. Nat Cell Biol 11 (8) 1002–1009. 19578371 10.1038/ncb1913 PMC2752141

